# Assessment of hypoxemia among young adults with sickle cell anaemia in steady state in southwestern Nigeria: a crosssectional study

**DOI:** 10.1186/s13104-024-06765-0

**Published:** 2024-04-08

**Authors:** Asafa M. A., Ahmed I. O, Afolabi M. S., Bolarinwa R. A., Ogunlade O.

**Affiliations:** 1 Department of Physiological Sciences, Faculty of Basic Medical Sciences, College of Health Sciences, Obafemi Awolowo University, Ile-Ife, Nigeria; 2 Department of Haematology and Blood Transfusion, Obafemi Awolowo University Teaching Hospital Complex, Ile-Ife, Nigeria; 3 Respiratory Unit, Department of Internal Medicine, Obafemi Awolowo University Teaching Hospital Complex, Ile-Ife, Nigeria; 4 Department of Haematology and Immunology, Faculty of Basic Medical Sciences, College of Health Sciences, Obafemi Awolowo University, Ile-Ife, Nigeria

**Keywords:** Hypoxemia, Sickle cell anaemia, Steady state, Vaso-occlusive crisis

## Abstract

**Objectives:**

Hypoxia is a known feature of sickle cell anaemia (SCA) which results from chronic anaemia and recurrent vaso-occlusive crisis (VOC) which can cause tissue ischaemia that leads to an end organ damage. The hallmark of SCA is chronic anaemia and recurrent vaso-occlusive crisis. The aim of this study is to compare the oxygen saturation of sickle cell anaemic individuals with the normal haemoglobin type (Hb AA) control and also to determine the prevalence of hypoxemia among SCA.

**Results:**

Two-hundred and twenty-two (136 Hb SS and 86 Hb AA) participated in the study. The mean ± SD of age (years), oxygen saturation (%) and pulse rate (bpm) of participants with sickle cell anaemia and Hb AA control were 21.85 ± 3.04 and 22.14 ± 3.18 (t = 0.701, *p* = 0.436), 95.21 ± 3.02 and 98.07 ± 0.81 (t=-8.598, *p* < 0.0001) and 77.10 ± 9.28 and 73.16 ± 8.52 (t = 3.173, *p* = 0.002) respectively. The prevalence of hypoxemia among SCA participants was 47.1%. Prevalence of hypoxemia in males with SCA was 60.9% while 39.1% of the females had hypoxemia.

## Introduction

Oxygen saturation (SaO_2_) is the representative of percentage of oxygen occupied on the binding sites on haemoglobin [1]. Adequate oxygen delivery to organs depends on effective oxygen transport to the tissue mitochondria, a critical task that is performed by haemoglobin and this is greatly reduced in anaemic state [2]. Reduced oxygen saturation results in inadequate tissue supply, particularly when oxygen demand is increased, as occurs during exercise [3]. Hypoxaemia is regarded as a low partial pressure of oxygen (PaO_2_) less than 8.0 kPa (60 mmHg) or peripheral arterial oxygen saturation (S_P_O_2_ ) less than 95% when measured by pulse oximetry [4, 5]. The (SaO_2_) of less than or equal to 95% is called hypoxaemia and it predicts a partial pressure of oxygen (PaO_2_) of less than 70 mm Hg based on a normal oxyhaemoglobin curve [6]. In SCA patients, a fall in SpO_2_ of 3% or more from baseline steady state values requires prompt intervention [7]. Campbell and Colleagues reported significantly lower SaO_2_ among adolescent with Hb SS when compared with Hb AA. This was said to result from lung disease that led to reduced gas exchange and alterations in the pulmonary vasculature or airways leading to ventilation-perfusion mismatching [8]. The significantly low SaO_2_ among individuals with SCA has been attributable to the chronic anaemia, microvascular occlusion of the circulation by sickled erythrocytes and constant perturbation of the endothelial membrane with consequent elaboration of endothelial molecules which are commonly seen among SCA children especially those with various types of vaso-occlusive crisis (VOC) [7, 9]. Lower SaO_2_ in people with SCA has also been reported to be associated with elevated serum lactate dehydrogenase, severe anaemia and reticulocytosis [10–12]. Previous studies among paediatric subjects have demonstrated high prevalence of hypoxemia among children with sickle cell anaemia [13, 14]. The improvement in healthcare of patients with SCA and the widespread adoption of comprehensive guidelines, the life expectancy of patients with SCA has risen, therefore, the prevalence of chronic organ dysfunction is also expected to increase [15, 16]. Few studies have assessed the prevalence of hypoxemia among adult SCA patients. The study determined the level of hypoxemia among the SCA compared SPO_2_ in young adults with SCA in steady state with age and sex matched individuals with HbAA.

## Methods

One hundred and thirty-six (67 males and 69 females) sickle cell anaemia individuals in steady state (group A) who were students of Obafemi Awolowo University, Ile-Ife, Nigeria participated in this study. The steady state was defined according Akinola et al. as the period free of crisis extending from at least three weeks since the last clinical event and three months or more since the last blood transfusion [17]. The inclusion criteria for the HbSS arm include age range of 18–40 years and willingness to participate in the study. The exclusion criteria were presence of ongoing crisis/ within the last one month, pregnancy, blood transfusion in the last three months and alcohol intake/ smoking within the last 4 weeks. Subjects with history of asthma were also excluded. The control group were 86 (42 males and 44 females) apparently healthy young adults with HbAA (group B) within the age range of 18–40 years who are age and sex-matched with group A, absence of systemic disease such as asthma and willingness to participate in the study. The SaO_2_ was determined using a pulse oximeter (ChoiceMMed_TM_). Non-invasive measurement of SpO_2_ was done by clipping the pulse oximeter to the left thumb after resting for five minutes in a thermoneutral environment. The SpO_2_ and heart rate were recorded after stabilization of the reading for one minute. This measurement was done twice and the average was calculated. The average SpO_2_ of less than or equal to 95% was considered as hypoxemia.

## Results

A total of 222 participants (136 HbSS and 86 HbAA) were recruited for this study. The mean ± SD age in years, SpO2 in % and pulse rate in beats per minutes of the Hb SS and Hb AA participants were 21.85 ± 3.04 and 22.14 ± 3.18 (t = 0.701, *p* = 0.436), 95.21 ± 3.02 and 98.07 ± 0.81 (t=-8.598, *p* < 0.0001) and 77.10 ± 9.28 and 73.16 ± 8.52 (t = 3.173, *p* = 0.002) respectively as shown in Table 1. Sixty-four (47.1%) of the participants with Hb SS had hypoxemia while none of the Hb AA participants had hypoxemia. Out of the 69 females with Hb SS that participated in the study, 27 (39.1%) had hypoxemia while 37 (55.2%) out of 67 males had hypoxemia as shown in Table 2.

**Table 1:** Effect of Haemoglobin type on Oxygen Saturation and Pulse Rate

	Hb SS (*n* = 136)	Hb AA (*n* = 86)	T	*p*-value
Age (years)	21.85 ± 3.04	22.14 ± 3.18	-0.701	0.436
Oxygen Saturation (%)	95.21 ± 3.02	98.07 ± 081	-8.598	< 0.001*
Pulse Rate (bpm)	77.10 ± 9.28	73.16 ± 8.52	3.173	0.002*

*- significant *p*-value

**Table 2:** Prevalence of Hypoxemia among Sickle Cell Anaemic Participants

	Hypoxemia	Non-hypoxemia
Male (*n* = 67)	37 (55.2%)	30 (44.8%)
Female (*n* = 69)	27 (39.1%)	42 (60.9%)
Total (*n* = 136)	64 (47.1%)	72 (52.9%)

Hypoxemia– SpO_2_ ≤ 95%, Non-hypoxemia– SpO_2_ > 95%. χ^2^ − 3.534, *p*-value– 0.061

## Discussion

The mean SpO_2_ of 95.21% among SCA observed in this study was similar to 95.5% reported by Ladu et al. among SCD patients who were within the same age range with our patients. Ladu et al. further explained that the low SpO_2_ among the SCA subjects were attributed to recurrent episodes of acute chest syndrome which results in a sequelae of irreversible chronic lung disease that gave rise to defective oxygenation of blood even at steady state [14]. Furthermore, haemoglobin S is known to have an inherent property of causing a rightward shift of the oxy-haemoglobin dissociation curve in an attempt to enhance oxygen delivery at the tissue level by raising level of 2,3-diphosphoglycerate in SCA rephrase this sentence to make it more concise [18, 19].

Ogunanobi et al. reported that the pulmonary function abnormality associated with SCD is as a result of membrane diffusion defects, intra pulmonary right-left shunts and shift of Hb oxygen dissociation curve to the right of normal [20]. Our findings revealed hypoxemia in 47.1% of SCA subjects in steady state which is higher than 13% reported by Chinawa et al. from a study comparing the prevalence of hypoxemia among children aged 6 months to 18 years with SCA during steady state and crises. None of their HBAA controls had hypoxemia which is exactly what we found in this study. The higher prevalence of hypoxemia in our study compared with findings from Chinawa et al. study may not be unconnected to the higher age of our subjects. In their study, hypoxemia was reportedly higher in the SCA cohort in crisis compared with those in steady state. This could be attributable to the chronic anaemic state and micro-vascular occlusion of the circulation by sickled red blood cells and pulmonary complications from recurrent sickling that is associated with VOC [13].

This study also showed a higher hypoxemia rate of 55.2% among male SCA subjects compared to 39.1% in the female cohort. Males SCA subjects have been reported to be prone to hypoxemia compared to their female counterparts, and this has been related to a lower transcription factor for hemoglobin F and Endothelial B receptor (ETBRT) in males. This receptor has been linked to X-chromosome and is found to be important in the body’s response to pain through the release of endorphins [21, 22]. Secondly, oestrogen is a potent vasodilator and can induce vascular relaxation by stimulating the release of endothelium-derived NO or by acting directly on the vascular smooth muscle (VSM) [23].

In conclusion, this study showed a high prevalence of hypoxemia in SCA patients in steady state, with males more affected. It is therefore recommended that steady state oxygen saturation should be established for all SCA patient while in their steady state and the interpretation of oxygen saturation should be individualized during sickle cell crisis.

## Data Availability

The datasets used and/or analysed during the current study are available from the corresponding author on reasonable request.
